# Serum Glycine Is Associated with Regional Body Fat and Insulin Resistance in Functionally-Limited Older Adults

**DOI:** 10.1371/journal.pone.0084034

**Published:** 2013-12-31

**Authors:** Michael S. Lustgarten, Lori Lyn Price, Edward M. Phillips, Roger A. Fielding

**Affiliations:** 1 Nutrition, Exercise Physiology, and Sarcopenia Laboratory, Jean Mayer USDA Human Nutrition Research Center, Tufts University, Boston, Massachusetts, United States of America; 2 The Institute for Clinical Research and Health Policy Studies, Tufts Medical Center, and Tufts Clinical and Translational Science Institute, Tufts University, Boston, Massachusetts, United States of America; University of Catanzaro Magna Graecia, Italy

## Abstract

**Background:**

Metabolic profiling may provide insight into biologic mechanisms related to age-related increases in regional adiposity and insulin resistance.

**Objectives:**

The objectives of the current study were to characterize the association between mid-thigh intermuscular and subcutaneous adipose tissue (IMAT, SCAT, respectively) and, abdominal adiposity with the serum metabolite profile, to identify significant metabolites as further associated with the homeostasis model assessment of insulin resistance (HOMA-IR), and, to develop a HOMA-IR associated metabolite predictor set representative of regional adiposity, in 73 functionally-limited (short physical performance battery ≤10; SPPB) older adults (age range, 70–85 y).

**Methods:**

Fasting levels of 181 total metabolites, including amino acids, fatty acids and acylcarnitines were measured with use of an untargeted mass spectrometry-based metabolomic approach. Multivariable-adjusted linear regression was used in all analyses.

**Results:**

Thirty-two, seven and one metabolite(s) were found to be associated with IMAT, abdominal adiposity and, SCAT, respectively, including the amino acid glycine, which was positively associated with SCAT and, negatively associated with both IMAT and abdominal adiposity. Glycine and four metabolites found to be significantly associated with regional adiposity were additionally associated with HOMA-IR. Separate stepwise regression models identified glycine as a HOMA-IR associated marker of both IMAT (model R^2^ = 0.51, p<0.0001) and abdominal adiposity (model R^2^ = 0.41, p<0.0001).

**Conclusion:**

Our findings for a positive association between glycine with SCAT but, a negative association between glycine with IMAT and abdominal adiposity supports the hypothesis that SCAT metabolic processes are different from that found in other fat depots. In addition, because of the significant associations found between glycine with HOMA-IR, IMAT, SCAT and abdominal adiposity, our results suggest glycine as a serum biomarker of both insulin sensitivity and regional fat mass in functionally-limited older adults.

## Introduction

Insulin resistance is well documented to occur with advancing age [1] and, is associated with an elevated risk of developing cardiovascular disease [2], [3], [4] and mortality [5]. One explanation for the increased prevalence of insulin resistance found in older adults involves elevated levels of total body adiposity [6]. However, not only the total amount of adipose tissue seems to be important, but also its distribution in the body [7]. For example, lower extremity intermuscular adipose tissue (IMAT) is elevated in older adults [8] and, is associated with insulin resistance [9], [10]. Similarly, age-related increases in abdominal adiposity may explain the increased incidence of insulin resistance found in older adults [11], [12], [13]. Interestingly, thigh IMAT and subcutaneous adipose tissue (SCAT) have been reported to have opposing effects on insulin sensitivity [14]. Thigh SCAT has previously been shown to be positively associated with insulin sensitivity, when examined independently of the other adipose tissue depots of the thigh [14], [15], and, elevated levels of thigh SCAT are independently associated with more favorable glucose and lipid profiles in overweight older adults [16]. Therefore, developing an improved understanding about mechanisms that underlie both regional adiposity and insulin resistance may lead to targeted interventions designed to reduce fat mass and to improve insulin sensitivity in older adults.

One approach that can be used to elucidate mechanisms related to both regional adiposity and insulin resistance is use of mass spectrometry-based (MS) metabolomics, which (when an untargeted approach is used) aims to characterize and quantify all of the small molecules in a sample (blood, urine, cerebrospinal fluid, etc.), thereby providing an analytical description of complex biological media [17]. Mass spectrometry-based (MS) metabolomic signatures have proven their value in several diseases, including diabetes and cardiovascular disease [18], and, cancer [19]. For example, MS-based metabolomic profiling of amino acids, fatty acids, and acylcarnitines [20] has provided support for the hypothesis that lipid-induced insulin resistance is explained in part by overload of mitochondrial lipid oxidation, accumulation of incompletely oxidized fats, and depletion of TCA intermediates, thereby leading to a condition of mitochondrial stress that activates signaling pathways that interfere with insulin action [21]. To date, only one study has investigated the association between circulating biomarkers (vitamins, adipokines and hormones) with regional adiposity in older adults (average age 63 y) [22], and, studies investigating the association between metabolic intermediates with both regional adiposity and insulin resistance in an older population have yet to be performed. Therefore, the goals of the present study were to characterize the association between serum MS metabolites with thigh IMAT, SCAT, and abdominal adiposity, to investigate the association between significant metabolites with HOMA-IR, and, to develop an insulin resistance-associated metabolite predictor set representative of regional fat mass, in functionally-limited older adults.

## Methods

### Ethics Statement

All participants signed an informed consent. Consent was confirmed by a witness to the process. Copies of signed consent were provided to all participants and originals were retained in study documents. All consent procedures and the study protocol were reviewed and approved by the Tufts University Health Sciences Campus Institutional Review Board.

### Participants

Serum from 73 (40 women, 30 men) overweight older adults (average BMI and age, 27.0 kg/m^2^, 77.8 y) with a demonstrated reduction in mobility (a score of 10 or less on the Short Physical Performance Battery, SPPB) from the randomized, double-blind, controlled study of Chale *et al*. (2012) [23] was subjected to analysis. All participants were required to be sedentary, defined as the absence of structured exercise during the previous 6 months. 28 of the 73 subjects in the current study were classified as insulin resistant (average HOMA-IR, 3.7), based on the HOMA-IR criteria defined by Stern *et al.* (2005) [24].

### Measurement of Regional Adiposity

Following an eight hour fast, thigh IMAT and SCAT were measured by computed tomography using a Siemens Somotom Scanner (Erlangen, Germany). Manual tracings were used to delineate thigh IMAT and SCAT in the range of −190 to −30 Hounsfield units (HU) using SliceOmatic v4.2 software (Montreal, Canada), as described previously [25]. Average measurement, standard error of measurement (SEM) and the intraclass correlation coefficient (ICC) for IMAT were 5.9 (range 3.2–9.3), 0.6 and 0.99 (95% CI 0.95, 1.0), respectively. Average measurement, SEM and ICC values for SCAT were 66.0 (range 26.4–138.4), 11.4 and 0.999 (95% CI 0.995, 1.0). To measure regional (arms, legs, trunk) and total fat mass, whole body dual-energy-x-ray absorptiometry scans were performed using a Hologic Discovery A densitometer. Scan acquisition and analysis were performed according to manufacturer guidelines, with three passes over the subject to acquire the full DXA image Scans were analyzed using Hologic QDR software version 12.3 in array mode (Hologic, Inc. Bedford, MA). Abdominal adiposity was determined via the trunk fat-periphery fat ratio, calculated by dividing the trunk fat mass by the sum of fat mass in the arms and legs [22].

### Measurement of HOMA-IR

Serum glucose was measured by the hexokinase method using a Beckman-Coulter AU400e chemistry system (Beckman-Coulter, Diagnostic Division, Brea, CA). Serum insulin was measured using a competitive binding radioimmunoassay (HI-14K Human Insulin Specific Kit, Millipore, St. Charles, MO). The Homeostasis Model Assessment of Insulin Resistance (HOMA-IR) is commonly used in large epidemiological studies and in clinical practice to estimate insulin resistance from fasting plasma insulin and glucose concentrations (Matthews 1985). HOMA-IR was calculated with the following formula: {(fasting serum insulin (mU/L) *x* fasting plasma glucose (mmol/L))}/22.5 [26].

### Metabolomic Analysis

Ten mL of blood was collected under standardized conditions between 8 and 10 AM and, following an overnight fast. After collection, blood was allowed to clot for one hour at room temperature, and, centrifuged at 2,135×*g* for 10 minutes at 4°C. Serum was derived by removing the supernatant following centrifugation and was stored in 1 mL aliquots at −80°C, prior to analysis.

Metabolomic data acquisition was performed by Metabolon Inc. (Research Triangle Park, NC). Briefly, small molecule metabolites were extracted from serum and the reconstituted extracts were resolved using mass spectrometry platforms, including ultrahigh performance liquid chromatography/tandem mass spectrometry (UHPLC/MS/MS) optimized for acidic and basic species, and, gas chromatography/mass spectrometry (GC/MS), with details of this platform described extensively by Evans *et al* [27]. Of the 296 metabolites that were identified, 181 metabolites were determined to be representative of fatty acids, amino acids, and acylcarnitines [20], [28], [29]. MS-based metabolomic profiling of amino acids, fatty acids, and acylcarnitines has previously been used to explain mechanisms related to lipid-induced insulin resistance [20], [21]. To examine the association between metabolites previously shown to be associated with insulin resistance with regional adiposity and HOMA-IR, serum data for amino acids, fatty acids, and acylcarnitines and, their related metabolites were used in multivariable statistical analyses.

### Statistics

Linear regression (SAS Enterprise Guide 4.3; SAS Institute Inc., Cary, NC) was used in multivariable analyses to determine the association between IMAT, SCAT and abdominal adiposity with serum metabolites, between significant metabolites with HOMA-IR, and, to identify an insulin resistance-associated metabolite predictor set representative of IMAT, SCAT and, abdominal adiposity.

Separate multivariable linear regression models were performed for each mass spectrometry-based metabolite. All regional fat models included sex, age, total fat and metabolite. HOMA-IR models included sex, age, total fat and metabolites found to be significantly associated with regional adiposity. Metabolite parameter estimates are based on a one-unit increase in the median scaled, natural log transformed metabolite data. False discovery rates [30] were computed with use of the q-value method [31] to adjust for multiple comparisons. Because conservatively chosen q-values may exclude biologically relevant associations, we chose to use a liberal q-value cutoff of 0.30 (and, p≤0.05), as reported by Meyers *et al* (2010) [32]. A q-value of 0.30 indicates that the result is likely to be valid 7 out of 10 times, which we suggest is reasonable in the setting of exploratory discovery.

To determine an insulin resistance-associated metabolite predictor set representative of IMAT and abdominal adiposity, sex, age and total fat-adjusted stepwise linear regression was used on metabolites that were found to be significantly associated with either IMAT or abdominal adiposity and, HOMA-IR. Because only one metabolite (glycine) was found to be associated with thigh subcutaneous adipose tissue, stepwise regression was not performed for SCAT. When stepwise selection is used, metabolites enter and remain in the model only if they are statistically significant (p≤0.05) after inclusion. Internal validation of the stepwise models was performed using a bootstrap re-sampling procedure of the dataset, using 200 individual bootstrap replicates. The pre-specified models were fitted to each, and an empiric 95% confidence interval (95% CI) was generated about the median of the 200 replicate parameter estimate values for each covariate.

## Results

General physical characteristics, including age, percent female, body weight, body mass index (BMI), total fat mass, trunk fat/peripheral fat, IMAT, SCAT and HOMA-IR are shown in Table 1.

**Table 1:** Subject demographics.

Age (y)	77.7±3.9
% Female	59
Body weight (kg)	74.0±10.6
BMI (kg/m^2^)	27.0±3.2
Total Fat Mass (kg)	25.6±7.2
Trunk Fat/Peripheral Fat	1.1±0.3
IMAT (cm^2^)	4.7**±**2.6
SCAT (cm^2^)	82.5±42.4
HOMA-IR	3.7±2.0

Values shown represent means±SD. IMAT, intermuscular adipose tissue; SCAT, subcutaneous adipose tissue; homeostasis model assessment of insulin resistance, HOMA-IR.

### Metabolites Associated with IMAT

Significant sex, age and total fat-adjusted associations between thirty-two metabolites with IMAT were identified, including eighteen amino acids and amino acid-related metabolites, eleven fatty acids and fatty acid-related metabolites, and, three carnitines (Table 2). Of the eighteen significant amino acids and amino acid-related metabolites, twelve metabolites were found to be positively associated with IMAT, including 2-hydroxyisobutryate, N-acetylthreonine, urea, 4-acetamidobutanoate, indolelactate, N-acetlyalanine, indoleacetate, symmetric dimethylarginine, γ-glutamylthreonine, N-methylproline, 2-hydroxy-3-methylvalerate and creatine. Six amino acids and amino acid-related metabolites were found to be negatively associated with IMAT, including glycine, glutamine, S-methylcysteine, serine, methionine and asparagine.

**Table 2:** Metabolites significantly associated with IMAT.

	β±SE	p-value	q-value
**Amino Acid Metabolism**			
2-hydroxyisobutyrate	2.5±0.6	1E-04	0.007
Glycine	−2.9±0.9	0.001	0.03
Glutamine	−5.1±1.7	0.003	0.04
N-acetylthreonine	2.5±0.8	0.003	0.04
Urea	1.6±0.6	0.007	0.07
S-methylcysteine	−1.7±0.6	0.01	0.07
4-acetamidobutanoate	2.2±0.9	0.01	0.07
Serine	−2.9±1.2	0.01	0.07
Methionine	−4.5±1.9	0.02	0.08
Indolelactate	1.5±0.7	0.03	0.10
N-acetylalanine	3.1±1.4	0.03	0.10
Indoleacetate	1.5±0.7	0.03	0.10
Symmetric dimethylarginine	2.5±1.2	0.03	0.10
γ-glutamylthreonine	1.4±0.7	0.04	0.10
N-methyl proline	0.5±0.2	0.05	0.10
2-hydroxy-3-methylvalerate	1.2±0.6	0.05	0.10
Asparagine	−2.5±1.2	0.05	0.10
Creatine	1.1±0.6	0.05	0.10
**Fatty Acid Metabolism**			
17-methylstearate	2.1±0.8	0.009	0.07
Dihomo-linolenate (20∶3n3 or n6)	2.4±0.9	0.01	0.07
Palmitate (16∶0)	2.9±1.1	0.01	0.07
Pentadecanoate (15∶0)	2.0±0.8	0.02	0.08
Docosadienoate (22∶2n6)	2.0±0.8	0.02	0.08
Margarate (17∶0)	1.8±0.8	0.02	0.08
10-heptadecenoate (17∶1n7)	1.7±0.8	0.03	0.10
Myristate (14∶0)	2.1±0.9	0.03	0.10
Docosapentaenoate (n6 DPA; 22∶5n6)	1.4±0.6	0.04	0.10
Stearidonate (18∶4n3)	1.3±0.6	0.04	0.10
Caproate (6∶0)	2.0±1.0	0.05	0.10
**Carnitine**			
Butyrylcarnitine	1.9±0.5	0.0006	0.02
3-dehydrocarnitine	2.4±0.8	0.003	0.04
Methylglutaroylcarnitine	0.6±0.3	0.04	0.10

All models were adjusted for sex, age and total fat. Metabolites are listed with their respective parameter estimates and standard errors (β±SE) in order of significance (p-value) and, with q-values.

All eleven fatty acids and fatty acid-related metabolites were found to be positively associated with IMAT, including saturated (caproate, C6∶0; myristate, C14∶0; pentadecanoate, C15∶0; palmitate, C16∶0, 17-methyl stearate, margarate, C17∶0), unsaturated (10-heptadecenoate, 17∶1n7) and polyunsaturated (stearidonate, 18∶4n3; dihomo-linolenate, 20∶3n3 or n6; docosadienoate, 22∶2n6; docosapentaenoate, n6 DPA; 22∶5n6) fatty acids.

Three carnitines were found to be significantly positively associated with IMAT, including butyrylcarnitine, 3-dehydrocarnitine and methylglutaroylcarnitine.

### Metabolites Associated with Abdominal Adiposity

Significant sex, age and total fat-adjusted associations between seven metabolites with abdominal adiposity were identified, including five amino acids and amino acid-related metabolites and, two carnitines (Table 3). Of the five significant amino acids and amino acid-related metabolites, positive associations between 2-hydroxy-3-methylvalerate and 3-hydroxyisobutyrate, and, negative associations between glycine, glutamine and 3-methoxtyrosine with abdominal adiposity were found. Among the carnitines, significant positive associations between isovalerylcarnitine and 3-dehydrocarnitine with abdominal adiposity were identified. Fatty acid metabolites were not found to be significantly associated with abdominal adiposity. Although ten additional metabolites (17-methylstearate, 2-hydroxybutyrate, α-hydroxyisovalerate, creatine, dihomo-linolenate (20∶3n3 or n6), palmitate, palmitate methyl-ester, serylleucine, trans-4-hydroxyproline, valine) were found to be significant at p≤0.05, q-values greater than 0.30 excluded them from statistical significance (*data not shown)*.

**Table 3:** Metabolites significantly associated with abdominal adiposity.

	β±SE	p-value	q-value
**Amino Acid Metabolism**			
Glycine	−0.3±0.1	0.002	0.15
2-hydroxy-3-methylvalerate	0.2±0.1	0.007	0.19
Glutamine	−0.5±0.2	0.008	0.19
3-hydroxyisobutyrate	0.3±0.1	0.007	0.19
3-methoxytyrosine	−0.3±0.1	0.01	0.24
**Carnitine**			
Isovalerylcarnitine	0.2±0.1	0.001	0.15
3-dehydrocarnitine	0.3±0.1	0.006	0.19

All models were adjusted for sex, age and total fat. Metabolites are listed with their respective parameter estimates and standard errors (β±SE) in order of significance (p-value) and, with q-values.

### Metabolites Associated with SCAT

In contrast with the significant negative associations found between glycine with both IMAT and abdominal adiposity, glycine was found to be significantly positively associated with SCAT (β±SE, 35.0±9.8, p = 0.0007, q = 0.13) after adjusting for sex, age and total fat. Although nine additional metabolites (2-hydroxy-3-methylvalerate, propionylcarnitine, butyrylcarnitine, 3-hydoxyisobutyrate, alanine, isovalerylcarnitine, leucylalanine, tiglylcarnitine, trans-4-hydroxyproline) were found to be significant at p≤0.05, q-values greater than 0.30 excluded them from statistical significance (*data not shown*). Interestingly, multiple metabolites found to be positively associated with either IMAT or abdominal adiposity (2-hydroxy-3-methylvalerate, butyrylcarnitne, isovalerylcarnine, 3-hydoxyisobutyrate) were found to be negatively associated (p≤0.05) with SCAT.

### Metabolites Associated with Regional Adiposity and HOMA-IR

Metabolites found to be associated with IMAT, abdominal adiposity, SCAT and, HOMA-IR are shown in Table 4. Glycine was found to be significantly negatively associated with HOMA-IR and, IMAT and abdominal adiposity. In contrast, a significant positive association between glycine with SCAT was identified. 2-hydroxy-3-methylvalerate was found to be significantly positively associated with HOMA-IR, IMAT and, abdominal adiposity. In contrast, a negative association (p≤0.05) between 2-hydroxy-3-methylvalerate with SCAT was identified, but a q-value greater than 0.3 (0.67) excluded it from statistical significance. Glutamine was found to be significantly negatively associated with HOMA-IR, IMAT and, abdominal adiposity. 2-hydroxyisobutryate and butyrylcarnitine were found to be significantly positively associated with HOMA-IR and IMAT. It is important to note that although butyrylcarnitine was found to be negatively associated with SCAT at p≤0.05, a q-value greater than 0.3 (0.67) excluded it from statistical significance.

**Table 4:** Metabolites associated with HOMA-IR, IMAT, abdominal adiposity, and, SCAT.

	HOMA-IR	IMAT	AbdominalAdiposity	SCAT
Glycine	−2.4±0.8*	−2.9±0.9*	−0.3±0.1*	35.0±9.8*
2-hydroxy-3-methylvalerate	1.8±0.5*	1.2±0.6*	0.2±0.1*	−16.4±6.7‡
Glutamine	−4.8±1.5*	−5.1±1.7*	−0.5±0.2*	23.7±19.9
2-hydroxyisobutyrate	1.3±0.6*	2.5±0.6*	0.0±0.1	−1.4±7.6
Butyrylcarnitine	1.5±0.5*	1.9±0.5*	0.1±0.1	−15.2±6.4‡

All models were adjusted for sex, age and total fat. Metabolites are listed with their respective parameter estimates and standard errors (β±SE).

p≤0.05, q ≤0.030;

p<0.05, q≥0.30.

### Stepwise Regression Identifies Glycine as a HOMA-IR Associated Predictor of IMAT and Abdominal Adiposity

To determine a HOMA-IR associated metabolite predictor set representative of IMAT and abdominal adiposity, multivariate-adjusted stepwise regression was used (Table 5). Although sex, age and total fat were found to explain 36.0% of the variability inherent in IMAT, the combination of glycine and 2-hydroxyisobutyrate were found to explain an additional 15%, for a total adjusted R^2^ of 51%. Similarly, although the combination of sex, age and total fat explained 33.4% of the variation inherent in abdominal adiposity, glycine was found to explain an additional 8%, for a total R^2^ of 41.4%. It is important to note that use of stepwise regression identified glycine as a negative predictor of both IMAT and abdominal adiposity. Furthermore, because glycine was the only metabolite significantly positively associated with thigh subcutaneous adipose tissue, stepwise regression for SCAT was not determined. To assess internal validity of the results obtained from stepwise regression bootstrapping was performed. 200 bootstrap samples were created, and the variables that were found to be significant in the stepwise models were forced in. The median R^2^ across the bootstrap replicates for the IMAT and abdominal adiposity models was found to be 0.55 (95% CI 0.38, 0.68) and 0.45 (95% CI 0.27, 0.57), respectively, which is similar to the model performance found in the stepwise models. Bootstrap-based 95% CI for the metabolites identified using stepwise regression are shown in Table 5.

**Table 5:** Stepwise linear regression on metabolites containing significant associations with regional adipose tissue and HOMA-IR.

	β±SE	p-value	Bootstrap β (95% CI)
**IMAT**			
Intercept	−17.3±4.9	0.0008	−16.7 (−26.3, −6.8)
Sex	1.2±0.5	0.03	1.6 (0.6, 2.8)
Age	0.2±0.1	0.002	0.2 (0.1, 0.3)
Total fat	0.2±0.0	2E-08	0.3 (0.2, 0.4)
2-hydroxyisobutyrate	2.0±0.9	0.002	2.0 (0.9, 3.0)
Glycine	−2.0±0.6	0.03	−1.9 (−3.5, −0.4)
**Abdominal Adiposity**			
Intercept	1.0±0.6	0.11	1.0 (−0.0, 2.2)
Sex	0.4±0.1	4E-07	0.4 (0.2, 0.5)
Age	−0.0±0.0	0.67	−0.0 (−0.0, 0.0)
Total fat	0.0±0.0	0.08	0.0 (−0.0, 0.0)
Glycine	−0.3±0.1	0.002	−0.3 (−0.5, −0.2)

All models were adjusted for sex, age and total fat. Covariates are listed with parameter estimates and standard errors (β**±**SE), and with p-values. Bootstrap parameter estimates (β) are shown with their respective 95% confidence intervals (CI).

## Discussion

The goals of the current study were to characterize the association between serum MS metabolites with thigh IMAT, SCAT, and abdominal adiposity, to investigate the association between significant metabolites with HOMA-IR, and, to develop a HOMA-IR-associated metabolite predictor set representative of regional adiposity, in functionally-limited older adults. Multivariable-adjusted linear regression on serum metabolomic data identified thirty-two, seven and one metabolite(s) to be significantly associated with IMAT, abdominal adiposity and SCAT, respectively, including the amino acid glycine, which was positively associated with SCAT and negatively associated with both IMAT and abdominal adiposity. Glycine and, four metabolites found to be significantly associated with regional adiposity were additionally associated with HOMA-IR. Multivariable-adjusted stepwise regression identified glycine as a HOMA-IR associated predictor of both IMAT and abdominal adiposity. In addition, glycine was the only metabolite to reach statistical significance in its association with SCAT, thereby identifying glycine as a HOMA-IR associated marker of multiple adipose-containing compartments. Because glycine has previously been shown to reduce plasma insulin and fat mass in rodents [33], [34], future studies aimed at testing the hypothesis that glycine supplementation may reduce fat mass and improve insulin sensitivity in glycine-deficient functionally-limited older adults are of interest.

Upon first inspection of the metabolites found to be associated with IMAT, two main trends were evident, including positive associations for all eleven fatty acids and, negative associations for five glucogenic amino acids. Elevated levels of plasma free fatty acids have been shown to reduce insulin-stimulated glucose uptake in both young healthy subjects [35], and, in those with type II diabetes [36]. Furthermore, we found significant positive associations for palmitate and dihomo-linolenate, fatty acids which are associated with peripheral insulin resistance in elderly men [37]. IMAT has been suggested to contribute to insulin resistance by enhancing rates of lipolysis within skeletal muscle [38], a finding that may explain the multiple associations found between serum fatty acids with IMAT. In addition, five glucogenic amino acids (asparagine, serine, glycine, glutamine, methionine) were found to be negatively associated with IMAT. Because the utilization of carbohydrate is somewhat impaired when insulin resistance is present, the oxidation of amino acids becomes an alternative energy source by entering the citric acid cycle at different points [39]. For example, asparagine is converted into oxaloacetate, serine and glycine are converted into pyruvate, glutamine is deaminated to yield α-ketoglutarate, and methionine yields succinyl-CoA [40]. Although prior evidence indicating these amino acids as markers of IMAT has yet to be reported, serum levels of glutamine and methionine were reduced in subjects with type II diabetes, relative to subjects with normal glucose tolerance [41]. Similarly, methionine, glutamine, asparagine, serine and glycine and were each reduced in plasma from 11-week old diabetic rats, relative to lean controls [42]. Collectively, our results suggest that the reduced levels of these amino acids previously found in association with insulin resistance and type II diabetes [41], [42] may be related to elevated levels of IMAT.

Separately, five amino acids and amino acid metabolites and, two carnitines were found to be significantly associated with abdominal adiposity. Interestingly, glycine and glutamine were negatively associated with both IMAT and abdominal adiposity, whereas three branched chain amino acid (BCAA)-related metabolites, 2-hydroxy-3-methylvalerate (formed during isoleucine degradation [43]), 3-hydroxyisobutyrate (formed during valine degradation [44]) and isovalerylcarnitine (the cognate acylcarnitine of isovaleryl-CoA, formed during leucine degradation [45]) were found to be positively associated with abdominal adiposity. Isoleucine deamination yields 2-oxo-3-methylvalerate [43]; reduction of this metabolite then produces 2-hydroxy-3-methylvalerate. Branched chain 2-keto dehydrogenase, the protein responsible for degradation of 2-oxo-3-methylvalerate has been shown to be reduced in adipose tissue from obese rodents [46]. The potential inability to degrade 2-oxo-3-methylvalerate may then lead to the accumulation of this metabolite, followed by mass action-driven reduction to produce 2-hydroxy-3-methylvalerate. Similarly, although 3-hydroxyisobutyrate and isovalerylcarnitine have yet to be reported as markers of abdominal adiposity, these metabolites are elevated in type II diabetics [47] and in obese, insulin resistant subjects (when compared with lean controls) [20]. As a possible explanation for these findings, multiple genes involved in BCAA catabolism have been shown to be down-regulated in adipose tissue from obese, when compared with non-obese subjects [48]. For example, isovaleryl CoA dehydrogenase gene expression is decreased in subcutaneous abdominal adipose tissue from insulin resistant, when compared with insulin sensitive subjects [49]. Collectively, these data suggest that mechanisms related to BCAA degradation in abdominal adipose tissue may be impaired in functionally-limited older adults.

In contrast with the negative associations found between glycine with both IMAT and abdominal adiposity, glycine was found to be positively associated with SCAT. In addition, the negative associations found between 2-hydroxy-3-methylvalerate and butyrylcarnitine with SCAT (found just outside of statistical significance, p≤0.05 but q ≥0.030), contrasts the positive associations found between these metabolites with either IMAT or abdominal adiposity. Furthermore, thigh SCAT was negatively associated (β±SE, −0.0±0.0, adjusted R^2^ = 0.21, p = 0.004), whereas both IMAT and abdominal adiposity were positively associated with HOMA-IR (IMAT β±SE, 0.3±0.1, adj. R^2^ = 0.19, p = 0.01; abdominal adiposity β±SE, 2.0±0.9, adj. R^2^ = 0.17, p = 0.03), after adjusting for age, sex and total fat. In agreement with these data, opposite associations for SCAT and IMAT with insulin sensitivity in obese HIV+, and, in healthy postmenopausal women, respectively, have been reported [14], [15]. As a potential explanation for the differences in insulin sensitivity found when comparing fat depots, adipocyte progenitor cells (APC) isolated from SCAT have intrinsic differences in gene expression, differentiation properties, and responses to environmental/genetic factors when compared with APC from visceral adipose tissue [50]. In addition, transplantation of SCAT from donor mice into visceral fat regions of recipient mice decreased body weight, total fat mass, glucose and insulin levels, evidence that suggests that SCAT produces substances that can act systemically to improve glucose metabolism [51]. Collectively, these data suggests that mechanisms related to glycine metabolism may underlie the metabolic differences inherent in thigh SCAT, when compared with thigh IMAT or abdominal adiposity.

Metabolites found to be significantly associated with regional adiposity were then further examined for their association with HOMA-IR, and, negative associations for both glycine and glutamine with HOMA-IR were identified. Glycine and glutamine have been reported to be negatively associated with HOMA-IR in subjects from the Framingham Heart Study [52]. Although concentrations of glycine in plasma and serum are reduced in insulin resistant, obese human subjects [20], [42], [53], our results are the first to more specifically associate glycine and glutamine with both HOMA-IR and, with IMAT, abdominal adiposity or SCAT. Furthermore, positive associations for 2-hydroxy-3-methylvalerate, 2-hydroxyisobutyrate and butyrylcarnitine with HOMA-IR were identified. 2-hydroxy-3-methylvalerate is a product of isoleucine degradation [43]. Isoleucine has been previously reported to be among an amino acid combination that is predictive of future diabetes risk [54]. Although isoleucine was not found to be associated with HOMA-IR or regional adiposity in the current study, our results additionally suggest the isoleucine degradation product 2-hydroxy-3-methylvalerate as an insulin resistance-associated marker of thigh IMAT and abdominal adiposity. Separately, increased urinary excretion of 2-hydroxyisobutyrate, derived from the microbial degradation of dietary proteins [55] has been reported in type 2 diabetic mice [56] and in obese humans [57]. Similarly, plasma levels of butyrylcarnitine were elevated in a rodent diabetes model, relative to controls [58], and have been shown to be elevated when mitochondrial fatty acid oxidation is impaired [59]. Collectively, these results suggest that mechanisms related to isoleucine degradation, gut microbial metabolism, and mitochondrial dysfunction may underlie both insulin resistance and regional adiposity, in functionally-limited older adults.

To determine an insulin resistance-associated metabolite predictor set representative of regional adiposity, we used multivariable-adjusted stepwise regression and found glycine (and 2-hydroxyisobutyrate, with IMAT) to be associated with both IMAT and abdominal adiposity, in separate models. Collectively, these data suggest glycine as a serum marker of both insulin sensitivity and, multiple adipose-containing compartments. Glycine’s impact on adiposity and insulin resistance can potentially be explained by improved insulin sensitivity [60], and/or increased antioxidative and anti-inflammatory capacity [61], [62]. We did not find an association between TNF-α or IL-6 with glycine in the current study (*data not shown*). However, in terms of the role of glycine on antioxidant capacity, glycine is a component of the tripeptide glutathione (GSH; comprised of glutamate, cysteine, glycine), the most abundant intracellular antioxidant [63]. If any of glutathione’s three amino acids are limiting, GSH synthesis will be suboptimal, potentially resulting in decreased insulin sensitivity [64]. In support of this hypothesis, insulin resistance is associated with a decreased cellular GSH concentration [65]. Dietary supplementation with a combination of cysteine plus glycine was sufficient to restore GSH to youthful levels and, improved insulin sensitivity, measured as a reduction in HOMA-IR in both old mice and in elderly humans, respectively [64]. Furthermore, glycine supplementation has been shown to reduce the increases in plasma insulin and intra-abdominal fat accumulation found when rats were fed a high sucrose diet [33], and, to decrease circulating concentrations of glucose, free fatty acids, triglycerides, adipocyte size and, adiposity in an animal model of intra-abdominal obesity [34]. Collectively, these studies suggest that randomized trials should be conducted to evaluate the impact of glycine supplementation on regional adiposity and insulin sensitivity in functionally-limited older adults.

In summary, although previous studies have identified various metabolites to be associated with either insulin resistance or whole-body adiposity, results obtained in the current study are the first attempt to associate serum metabolic intermediates with both HOMA-IR and regional adiposity, including thigh IMAT and SCAT, and, abdominal adiposity. The main finding of the current study is that the amino amino acid glycine is a HOMA-IR associated marker of IMAT, SCAT and, abdominal adiposity. Future studies aimed at testing the hypothesis that glycine supplementation will reduce fat mass and improve insulin sensitivity in glycine-deficient functionally-limited older adults are of interest.

## References

[1] Barbieri M, Rizzo MR, Manzella D, Paolisso G (2001) Age-related insulin resistance: is it an obligatory finding? The lesson from healthy centenarians. Diabetes Metab Res Rev 17: 19–26. 11241888 10.1002/dmrr.178

[2] Bonora E, Kiechl S, Willeit J, Oberhollenzer F, Egger G, et al (2007) Insulin resistance as estimated by homeostasis model assessment predicts incident symptomatic cardiovascular disease in caucasian subjects from the general population: the Bruneck study. Diabetes Care 30: 318–324. 17259501 10.2337/dc06-0919

[3] Ginsberg HN (2000) Insulin resistance and cardiovascular disease. J Clin Invest 106: 453–458. 10953019 10.1172/JCI10762 PMC380256

[4] Hanley AJ, Williams K, Stern MP, Haffner SM (2002) Homeostasis model assessment of insulin resistance in relation to the incidence of cardiovascular disease: the San Antonio Heart Study. Diabetes Care 25: 1177–1184. 12087016 10.2337/diacare.25.7.1177

[5] Noale M, Maggi S, Zanoni S, Limongi F, Zambon S, et al (2012) The metabolic syndrome, incidence of diabetes and mortality among the elderly: the Italian Longitudinal Study of Ageing. Diabetes Metab 38: 135–141. 22071281 10.1016/j.diabet.2011.09.005

[6] Lim SC, Tan BY, Chew SK, Tan CE (2002) The relationship between insulin resistance and cardiovascular risk factors in overweight/obese non-diabetic Asian adults: the 1992 Singapore National Health Survey. Int J Obes Relat Metab Disord 26: 1511–1516. 12439654 10.1038/sj.ijo.0802140

[7] Smith SR, Lovejoy JC, Greenway F, Ryan D, deJonge L, et al (2001) Contributions of total body fat, abdominal subcutaneous adipose tissue compartments, and visceral adipose tissue to the metabolic complications of obesity. Metabolism 50: 425–435. 11288037 10.1053/meta.2001.21693

[8] Delmonico MJ, Harris TB, Visser M, Park SW, Conroy MB, et al (2009) Longitudinal study of muscle strength, quality, and adipose tissue infiltration. Am J Clin Nutr 90: 1579–1585. 19864405 10.3945/ajcn.2009.28047 PMC2777469

[9] Goodpaster BH, Krishnaswami S, Resnick H, Kelley DE, Haggerty C, et al (2003) Association between regional adipose tissue distribution and both type 2 diabetes and impaired glucose tolerance in elderly men and women. Diabetes Care 26: 372–379. 12547865 10.2337/diacare.26.2.372

[10] Goodpaster BH, Thaete FL, Kelley DE (2000) Thigh adipose tissue distribution is associated with insulin resistance in obesity and in type 2 diabetes mellitus. Am J Clin Nutr 71: 885–892. 10731493 10.1093/ajcn/71.4.885

[11] Kohrt WM (1998) Abdominal obesity and associated cardiovascular comorbidities in the elderly. Coron Artery Dis 9: 489–494. 9847980 10.1097/00019501-199809080-00004

[12] Kohrt WM, Kirwan JP, Staten MA, Bourey RE, King DS, et al (1993) Insulin resistance in aging is related to abdominal obesity. Diabetes 42: 273–281. 8425663

[13] Shimokata H, Tobin JD, Muller DC, Elahi D, Coon PJ, et al (1989) Studies in the distribution of body fat: I. Effects of age, sex, and obesity. J Gerontol 44: M66–73. 2921472 10.1093/geronj/44.2.m66

[14] Goss AM, Gower BA (2012) Insulin sensitivity is associated with thigh adipose tissue distribution in healthy postmenopausal women. Metabolism 61: 1817–1823. 22748969 10.1016/j.metabol.2012.05.016 PMC3465478

[15] Albu JB, Kenya S, He Q, Wainwright M, Berk ES, et al (2007) Independent associations of insulin resistance with high whole-body intermuscular and low leg subcutaneous adipose tissue distribution in obese HIV-infected women. Am J Clin Nutr 86: 100–106. 17616768 10.1093/ajcn/86.1.100 PMC2670485

[16] Snijder MB, Visser M, Dekker JM, Goodpaster BH, Harris TB, et al (2005) Low subcutaneous thigh fat is a risk factor for unfavourable glucose and lipid levels, independently of high abdominal fat. The Health ABC Study. Diabetologia 48: 301–308. 15660262 10.1007/s00125-004-1637-7

[17] Fiehn O (2002) Metabolomics–the link between genotypes and phenotypes. Plant Mol Biol 48: 155–171. 11860207

[18] Magnusson M, Lewis GD, Ericson U, Orho-Melander M, Hedblad B, et al.. (2012) A diabetes-predictive amino acid score and future cardiovascular disease. Eur Heart J. 10.1093/eurheartj/ehs424 PMC3703309 23242195

[19] Yang J, Xu G, Zheng Y, Kong H, Pang T, et al (2004) Diagnosis of liver cancer using HPLC-based metabonomics avoiding false-positive result from hepatitis and hepatocirrhosis diseases. J Chromatogr B Analyt Technol Biomed Life Sci 813: 59–65. 10.1016/j.jchromb.2004.09.032 15556516

[20] Newgard CB, An J, Bain JR, Muehlbauer MJ, Stevens RD, et al (2009) A branched-chain amino acid-related metabolic signature that differentiates obese and lean humans and contributes to insulin resistance. Cell Metab 9: 311–326. 19356713 10.1016/j.cmet.2009.02.002 PMC3640280

[21] Bain JR, Stevens RD, Wenner BR, Ilkayeva O, Muoio DM, et al (2009) Metabolomics applied to diabetes research: moving from information to knowledge. Diabetes 58: 2429–2443. 19875619 10.2337/db09-0580 PMC2768174

[22] Lim U, Turner SD, Franke AA, Cooney RV, Wilkens LR, et al (2012) Predicting total, abdominal, visceral and hepatic adiposity with circulating biomarkers in Caucasian and Japanese American women. PLoS One 7: e43502. 22912885 10.1371/journal.pone.0043502 PMC3422255

[23] Chale A, Cloutier GJ, Hau C, Phillips EM, Dallal GE, et al.. (2012) Efficacy of Whey Protein Supplementation on Resistance Exercise-Induced Changes in Lean Mass, Muscle Strength, and Physical Function in Mobility-Limited Older Adults. J Gerontol A Biol Sci Med Sci. 10.1093/gerona/gls221 PMC3708517 23114462

[24] Stern SE, Williams K, Ferrannini E, DeFronzo RA, Bogardus C, et al (2005) Identification of individuals with insulin resistance using routine clinical measurements. Diabetes 54: 333–339. 15677489 10.2337/diabetes.54.2.333

[25] Frontera WR, Reid KF, Phillips EM, Krivickas LS, Hughes VA, et al (2008) Muscle fiber size and function in elderly humans: a longitudinal study. J Appl Physiol 105: 637–642. 18556434 10.1152/japplphysiol.90332.2008 PMC2519941

[26] Matthews DR, Hosker JP, Rudenski AS, Naylor BA, Treacher DF, et al (1985) Homeostasis model assessment: insulin resistance and beta-cell function from fasting plasma glucose and insulin concentrations in man. Diabetologia 28: 412–419. 3899825 10.1007/BF00280883

[27] Evans AM, DeHaven CD, Barrett T, Mitchell M, Milgram E (2009) Integrated, nontargeted ultrahigh performance liquid chromatography/electrospray ionization tandem mass spectrometry platform for the identification and relative quantification of the small-molecule complement of biological systems. Anal Chem 81: 6656–6667. 19624122 10.1021/ac901536h

[28] Ha CY, Kim JY, Paik JK, Kim OY, Paik YH, et al (2012) The association of specific metabolites of lipid metabolism with markers of oxidative stress, inflammation and arterial stiffness in men with newly diagnosed type 2 diabetes. Clin Endocrinol (Oxf) 76: 674–682. 21958081 10.1111/j.1365-2265.2011.04244.x

[29] Wu T, Xie C, Han J, Ye Y, Weiel J, et al (2012) Metabolic disturbances associated with systemic lupus erythematosus. PLoS One 7: e37210. 22723834 10.1371/journal.pone.0037210 PMC3378560

[30] Benjamini Y HY (1995) Controlling the false discovery rate: A practical and powerful approach to multiple testing. Journal of the Royal Statistical Society, Series B 57: 289–300.

[31] Storey JD, Tibshirani R (2003) Statistical significance for genomewide studies. Proc Natl Acad Sci U S A 100: 9440–9445. 12883005 10.1073/pnas.1530509100 PMC170937

[32] Meyers KJ, Chu J, Mosley TH, Kardia SL (2010) SNP-SNP interactions dominate the genetic architecture of candidate genes associated with left ventricular mass in African-Americans of the GENOA study. BMC Med Genet 11: 160. 21067599 10.1186/1471-2350-11-160 PMC2991303

[33] El Hafidi M, Perez I, Zamora J, Soto V, Carvajal-Sandoval G, et al (2004) Glycine intake decreases plasma free fatty acids, adipose cell size, and blood pressure in sucrose-fed rats. Am J Physiol Regul Integr Comp Physiol 287: R1387–1393. 15331379 10.1152/ajpregu.00159.2004

[34] Alvarado-Vasquez N, Zamudio P, Ceron E, Vanda B, Zenteno E, et al (2003) Effect of glycine in streptozotocin-induced diabetic rats. Comp Biochem Physiol C Toxicol Pharmacol 134: 521–527. 12727302 10.1016/s1532-0456(03)00046-2

[35] Boden G (2001) Pathogenesis of type 2 diabetes. Insulin resistance. Endocrinol Metab Clin North Am 30: 801–815, v. 10.1016/s0889-8529(05)70216-4 11727400

[36] Boden G, Chen X (1995) Effects of fat on glucose uptake and utilization in patients with non-insulin-dependent diabetes. J Clin Invest 96: 1261–1268. 7657800 10.1172/JCI118160 PMC185747

[37] Vessby B, Tengblad S, Lithell H (1994) Insulin sensitivity is related to the fatty acid composition of serum lipids and skeletal muscle phospholipids in 70-year-old men. Diabetologia 37: 1044–1050. 7851683 10.1007/BF00400468

[38] Maggs DG, Jacob R, Rife F, Lange R, Leone P, et al (1995) Interstitial fluid concentrations of glycerol, glucose, and amino acids in human quadricep muscle and adipose tissue. Evidence for significant lipolysis in skeletal muscle. J Clin Invest 96: 370–377. 7615807 10.1172/JCI118043 PMC185209

[39] Chevalier S, Burgess SC, Malloy CR, Gougeon R, Marliss EB, et al (2006) The greater contribution of gluconeogenesis to glucose production in obesity is related to increased whole-body protein catabolism. Diabetes 55: 675–681. 16505230 10.2337/diabetes.55.03.06.db05-1117

[40] Ferrier DR, editor (2013) Biochemistry (Lippincott's Illustrated Reviews Series). Sixth ed. Hagerstwon: Lippincott Williams & Wilkins. 560 p.

[41] Zhang D, Liu ZX, Choi CS, Tian L, Kibbey R, et al (2007) Mitochondrial dysfunction due to long-chain Acyl-CoA dehydrogenase deficiency causes hepatic steatosis and hepatic insulin resistance. Proc Natl Acad Sci U S A 104: 17075–17080. 17940018 10.1073/pnas.0707060104 PMC2040460

[42] Wijekoon EP, Skinner C, Brosnan ME, Brosnan JT (2004) Amino acid metabolism in the Zucker diabetic fatty rat: effects of insulin resistance and of type 2 diabetes. Can J Physiol Pharmacol 82: 506–514. 15389298 10.1139/y04-067

[43] Mamer OA, Reimer ML (1992) On the mechanisms of the formation of L-alloisoleucine and the 2-hydroxy-3-methylvaleric acid stereoisomers from L-isoleucine in maple syrup urine disease patients and in normal humans. J Biol Chem 267: 22141–22147. 1429566

[44] Murin R, Schaer A, Kowtharapu BS, Verleysdonk S, Hamprecht B (2008) Expression of 3-hydroxyisobutyrate dehydrogenase in cultured neural cells. J Neurochem 105: 1176–1186. 18284611 10.1111/j.1471-4159.2008.05298.x

[45] McFarlane IG, Von Holt C (1969) Metabolism of amino acids in protein-calorie-deficient rats. Biochem J 111: 557–563. 5774479 10.1042/bj1110557 PMC1187576

[46] She P, Van Horn C, Reid T, Hutson SM, Cooney RN, et al (2007) Obesity-related elevations in plasma leucine are associated with alterations in enzymes involved in branched-chain amino acid metabolism. Am J Physiol Endocrinol Metab 293: E1552–1563. 17925455 10.1152/ajpendo.00134.2007 PMC2767201

[47] Avogaro A, Bier DM (1989) Contribution of 3-hydroxyisobutyrate to the measurement of 3-hydroxybutyrate in human plasma: comparison of enzymatic and gas-liquid chromatography-mass spectrometry assays in normal and in diabetic subjects. J Lipid Res 30: 1811–1817. 2614280

[48] Pietilainen KH, Naukkarinen J, Rissanen A, Saharinen J, Ellonen P, et al (2008) Global transcript profiles of fat in monozygotic twins discordant for BMI: pathways behind acquired obesity. PLoS Med 5: e51. 18336063 10.1371/journal.pmed.0050051 PMC2265758

[49] Elbein SC, Kern PA, Rasouli N, Yao-Borengasser A, Sharma NK, et al (2011) Global gene expression profiles of subcutaneous adipose and muscle from glucose-tolerant, insulin-sensitive, and insulin-resistant individuals matched for BMI. Diabetes 60: 1019–1029. 21266331 10.2337/db10-1270 PMC3046820

[50] Macotela Y, Emanuelli B, Mori MA, Gesta S, Schulz TJ, et al (2012) Intrinsic differences in adipocyte precursor cells from different white fat depots. Diabetes 61: 1691–1699. 22596050 10.2337/db11-1753 PMC3379665

[51] Tran TT, Yamamoto Y, Gesta S, Kahn CR (2008) Beneficial effects of subcutaneous fat transplantation on metabolism. Cell Metab 7: 410–420. 18460332 10.1016/j.cmet.2008.04.004 PMC3204870

[52] Cheng S, Rhee EP, Larson MG, Lewis GD, McCabe EL, et al (2012) Metabolite profiling identifies pathways associated with metabolic risk in humans. Circulation 125: 2222–2231. 22496159 10.1161/CIRCULATIONAHA.111.067827 PMC3376658

[53] Felig P, Marliss E, Cahill GF Jr (1969) Plasma amino acid levels and insulin secretion in obesity. N Engl J Med 281: 811–816. 5809519 10.1056/NEJM196910092811503

[54] Wang TJ, Larson MG, Vasan RS, Cheng S, Rhee EP, et al (2011) Metabolite profiles and the risk of developing diabetes. Nat Med 17: 448–453. 21423183 10.1038/nm.2307 PMC3126616

[55] Li M, Wang B, Zhang M, Rantalainen M, Wang S, et al (2008) Symbiotic gut microbes modulate human metabolic phenotypes. Proc Natl Acad Sci U S A 105: 2117–2122. 18252821 10.1073/pnas.0712038105 PMC2538887

[56] Connor SC, Hansen MK, Corner A, Smith RF, Ryan TE (2010) Integration of metabolomics and transcriptomics data to aid biomarker discovery in type 2 diabetes. Mol Biosyst 6: 909–921. 20567778 10.1039/b914182k

[57] Friedrich N, Budde K, Wolf T, Jungnickel A, Grotevendt A, et al (2012) Short-term changes of the urine metabolome after bariatric surgery. OMICS 16: 612–620. 23095112 10.1089/omi.2012.0066

[58] Chen KH, Cheng ML, Jing YH, Chiu DT, Shiao MS, et al (2011) Resveratrol ameliorates metabolic disorders and muscle wasting in streptozotocin-induced diabetic rats. Am J Physiol Endocrinol Metab 301: E853–863. 21791624 10.1152/ajpendo.00048.2011

[59] Jethva R, Bennett MJ, Vockley J (2008) Short-chain acyl-coenzyme A dehydrogenase deficiency. Mol Genet Metab 95: 195–200. 18977676 10.1016/j.ymgme.2008.09.007 PMC2720545

[60] Gannon MC, Nuttall JA, Nuttall FQ (2002) The metabolic response to ingested glycine. Am J Clin Nutr 76: 1302–1307. 12450897 10.1093/ajcn/76.6.1302

[61] Cruz M, Maldonado-Bernal C, Mondragon-Gonzalez R, Sanchez-Barrera R, Wacher NH, et al (2008) Glycine treatment decreases proinflammatory cytokines and increases interferon-gamma in patients with type 2 diabetes. J Endocrinol Invest 31: 694–699. 18852529 10.1007/BF03346417

[62] Sekhar RV, McKay SV, Patel SG, Guthikonda AP, Reddy VT, et al (2011) Glutathione synthesis is diminished in patients with uncontrolled diabetes and restored by dietary supplementation with cysteine and glycine. Diabetes Care 34: 162–167. 20929994 10.2337/dc10-1006 PMC3005481

[63] Deneke SM, Fanburg BL (1989) Regulation of cellular glutathione. Am J Physiol 257: L163–173. 2572174 10.1152/ajplung.1989.257.4.L163

[64] Nguyen D, Samson SL, Reddy VT, Gonzalez EV, Sekhar RV (2013) Impaired mitochondrial fatty acid oxidation and insulin resistance in aging: novel protective role of glutathione. Aging Cell. 10.1111/acel.12073 23534396

[65] Memisogullari R, Taysi S, Bakan E, Capoglu I (2003) Antioxidant status and lipid peroxidation in type II diabetes mellitus. Cell Biochem Funct 21: 291–296. 12910484 10.1002/cbf.1025

